# N- and L-Type Voltage-Gated Calcium Channels Mediate Fast Calcium Transients in Axonal Shafts of Mouse Peripheral Nerve

**DOI:** 10.3389/fncel.2016.00135

**Published:** 2016-06-02

**Authors:** Ruxandra Barzan, Friederike Pfeiffer, Maria Kukley

**Affiliations:** Group of Neuron Glia Interaction, Werner Reichardt Centre for Integrative Neuroscience, University of TübingenTübingen, Germany

**Keywords:** Ca^2+^ channels, peripheral nervous system, axonal shafts

## Abstract

In the peripheral nervous system (PNS) a vast number of axons are accommodated within fiber bundles that constitute peripheral nerves. A major function of peripheral axons is to propagate action potentials along their length, and hence they are equipped with Na^+^ and K^+^ channels, which ensure successful generation, conduction and termination of each action potential. However little is known about Ca^2+^ ion channels expressed along peripheral axons and their possible functional significance. The goal of the present study was to test whether voltage-gated Ca^2+^ channels (VGCCs) are present along peripheral nerve axons *in situ* and mediate rapid activity-dependent Ca^2+^ elevations under physiological circumstances. To address this question we used mouse sciatic nerve slices, Ca^2+^ indicator Oregon Green BAPTA-1, and 2-photon Ca^2+^ imaging in fast line scan mode (500 Hz). We report that transient increases in intra-axonal Ca^2+^ concentration take place along peripheral nerve axons *in situ* when axons are stimulated electrically with single pulses. Furthermore, we show for the first time that Ca^2+^ transients in peripheral nerves are fast, i.e., occur in a millisecond time-domain. Combining Ca^2+^ imaging and pharmacology with specific blockers of different VGCCs subtypes we demonstrate that Ca^2+^ transients in peripheral nerves are mediated mainly by N-type and L-type VGCCs. Discovery of fast Ca^2+^ entry into the axonal shafts through VGCCs in peripheral nerves suggests that Ca^2+^ may be involved in regulation of action potential propagation and/or properties in this system, or mediate neurotransmitter release along peripheral axons as it occurs in the optic nerve and white matter of the central nervous system (CNS).

## Introduction

In the peripheral nervous system (PNS) a vast number of axons are accommodated within fiber bundles that constitute peripheral nerves. The major function of peripheral axons is to propagate action potentials along their length, therefore axons are equipped with voltage-gated Na^+^ and K^+^ channels which ensure successful generation, conduction and termination of each action potential. In addition to Na^+^ and K^+^ channels voltage-gated Ca^2+^ channels (VGCCs) are expressed on peripheral axons. However only few groups have so far directly studied these channels and very little is known about their sub-types, developmental regulation, and function. Ca^2+^-conductance probably mediated by VGCCs was detected in rat preganglionic cervical sympathetic nerves (Elliott et al., 1989) and in unmyelinated fibers of biopsied human sural nerve (Quasthoff et al., 1995, 1996). Increase in intra-axonal Ca^2+^ level along peripheral axons was reported during prolonged (0.3–10 s) repetitive electrical stimulation of rat vagus nerve and of biopsied human sural nerves (Wächtler et al., 1998; Mayer et al., 1999), yet the exact channel subtypes mediating Ca^2+^ influx remain unknown. In mouse postganglionic sympathetic axonal bundles Ca^2+^ transients could be detected not only during train stimulation but also in response to a single stimulus (Jackson et al., 2001). VGCCs located along axonal shafts in the PNS could be of great significance for modulation of action potential conduction velocity and/or frequency (François et al., 2015), fast axonal transport (Chan et al., 1980), or release of neuropeptides (Eberhardt et al., 2008; Spitzer et al., 2008). To play a modulatory role during these fast cellular processes, Ca^2+^ transients in the peripheral axons should occur in a millisecond time domain. Yet, it remains unclear whether rapid activity-dependent Ca^2+^ elevations take place along peripheral axons under physiological conditions, because in the previous studies either image acquisition has been done using relatively slow frame scanning mode and low sampling rate (Wächtler et al., 1998; Mayer et al., 1999; Jackson et al., 2001), or Ca^2+^ conductance has been measured with blockers of K^+^ channels in the bath or strongly elevated extracellular K^+^ concentration (Elliott et al., 1989; Quasthoff et al., 1995, 1996). It is also un-clear which types of VGCCs mediate rapid Ca^2+^ elevations in the peripheral axons. Remarkably, in the central nervous system (CNS) VGCCs are present along the axons in several structures including retina (Sargoy et al., 2014), cerebellum (Callewaert et al., 1996; Forti et al., 2000), corpus callosum (Kukley et al., 2007), optic nerve (Lev-Ram and Grinvald, 1987; Fern et al., 1995; Sun and Chiu, 1999; Brown et al., 2001; Zhang et al., 2006; Alix et al., 2008) and spinal dorsal column (Ouardouz et al., 2003). They open in a millisecond time domain upon action potential arrival and mediate fast Ca^2+^ transients which are similar to those observed in presynaptic nerve terminals at conventional neuronal synapses (Lev-Ram and Grinvald, 1987; Sun and Chiu, 1999; Kukley et al., 2007). Axonal Ca^2+^ transients in the CNS are involved in synaptic signaling between axons and oligodendrocyte progenitor cells (Kukley et al., 2007; Ziskin et al., 2007), modulation of axonal excitability, and regulation of intracellular Ca^2+^ level during axonal growth (Sun and Chiu, 1999; Bucher and Goaillard, 2011).

A major goal of the present study was to test whether VGCCs mediate rapid (in a millisecond time domain) activity-dependent Ca^2+^ elevations along mammalian peripheral nerve axons *in situ* under physiological conditions. Answering this question is of great importance for the follow-up research on the functional role of VGCCs in peripheral nerves *in situ* and *in vivo*, and is also of clinical and pharmaceutical relevance. Using 2-photon Ca^2+^ imaging in line scan mode we found that action potentials trigger fast Ca^2+^ transients along peripheral nerve axons *in situ*; these Ca^2+^ transients involve activation of N- and-L-type VGCCs.

## Materials and Methods

### Animals

C57BL/6N mice were originally obtained from Charles River and bred in house. All experiments were performed in accordance with the guidelines of the Animal Care and Use Committee at the University of Tübingen.

### Preparation of Sciatic Nerve Live Slices

Newborn mouse pups (P0–2) were sacrificed by decapitation without anesthesia and both sciatic nerves were isolated. The nerves were transferred to a Petri dish and maintained for ~15 min in ice-cold high-Mg^2+^ ACSF containing in mM: 124 NaCl, 1.25 NaH_2_PO_4_, 10 MgSO_4_, 2.7 KCl, 26 NaHCO_3_, 2 CaCl_2_, 2 ascorbic acid, 18.6 glucose. Subsequently nerves were embedded into 2.5% low-melting agarose dissolved in normal ACSF, containing in mM: 124 NaCl, 1.25 NaH_2_PO_4_, 1.3 MgSO_4_, 2.7 KCl, 26 NaHCO_3_, 2 CaCl_2_, 2 ascorbic acid, 18.6 glucose and cooled down to 37°C. One hundred micrometres-thin longitudinal nerve slices were prepared on a vibratome (VT1200S, Leica Biosystems), using ice-cold high-Mg^2+^ACSF. The slices were placed into a Haas-type interface chamber and maintained at room temperature up to 8 h perfused with normal ACSF gassed with 95% O_2_ and 5% CO_2_.

### Ca^2+^ Indicator Injection

Individual sciatic nerve slices were loaded with a high-affinity Ca^2+^ indicator Oregon-green-BAPTA-1 (OGB-1 AM) or low-affinity indicator Magnesium Green, as described previously (Regehr, 2000). Briefly, 50 μg of Ca^2+^ indicator was dissolved in 20 μl of pluronic acid in dimethyl sulfoxide (20% w/v). Four hundred microliter normal ACSF was added to this solution. The final Ca^2+^ indicator concentration was ~100 μM. A volume of 5 μl of the indicator solution was loaded into a glass micropipette (diameter ~3–6 μm) which was lowered into a nerve slice; a small positive pressure was applied for 10–15 min. Subsequently the slice was washed with ACSF for ~15 min.

### Ca^2+^ Imaging with Two-Photon Excitation Microscopy

Individual nerve slices filled with Ca^2+^ indicator were transferred to a recording chamber mounted on the stage of a 2-photon laser-scanning microscope (LaVision Biotech, Germany) and perfused with ACSF containing 2.5 mM Ca^2+^. Axons were stimulated with a monopolar glass electrode (3–6 μm tip diameter) filled with normal ASCF. Single pulses (pulse length 200–500 μs, pulse amplitude 50 V) were applied every 30 s using isolated pulse stimulator (ISO-STIM 01D, NPI Electronic, Germany). To acquire high temporal resolution, line scanning was performed perpendicular to the orientation of the axons, with a frequency of 500 Hz. The dye was excited at 790 nm (Spectra-Physics MaiTai HP Laser) and fluorescence signals were detected using a high sensitivity photomultiplier H7422-40 (Hamamatsu, Japan), after filtering with a DCLP dichroic mirror >500 nm. A laser-scanning system (TriM Scope II, LaVisionBiotec, Germany) coupled to an upright microscope (Olympus, Japan) equipped with a 20×, NA 1.1 water-immersion objective (Zeiss, Germany) was controlled using ImSpector Pro Software (version 4.0, LaVision Biotec, Germany), which also allowed online analysis of the data. The scan head and stimulator were synchronized using Igor Pro 6.2 Software (WaveMetrics, Lake Oswego, OR, USA) and an external trigger system (SyncUnit, LaVisionBiotec, Germany).

### Analysis of Ca^2+^ Imaging Data

The amplitude of the Ca^2+^ fluorescence signal was measured in parts of axonal bundles positioned in the focal plane, as the ratio of the difference between the peak fluorescence and the resting fluorescence (ΔF = F−F_0_) and the resting fluorescence (F_0_), after background subtraction. Background region was chosen as the less bright area in the field of view (FOV; not more than 10 μm away from the recorded axon). The analysis was performed using custom-written macros for IgorPro (WaveMetrics, Lake Oswego, OR, USA). 10–90% rise-time of Ca^2+^ transients was measured manually using hairline cursors in IgorPro. To determine the decay time constant, a mono-exponential function was fitted to the decaying part of the transient, from the peak until ~500 ms after the peak. The graphs show mean ± standard error (SEM).

### Measurement of Axon Diameter

To estimate the diameter of single axons within small axonal bundles from which Ca^2+^ transients were recorded, 3D line-scan pictures (*z*-stack) of the axons loaded with OGB-1 were recorded. The brightness profile of the line-scans was plotted, and the diameter of single axons was measured in the plane where the axons were in focus. ImSpector Pro Software (LaVision Biotech, Germany) was employed for these measurements.

### Immunohistochemistry and Confocal Laser Scanning Microscopy

Newborn mouse pups (P0–2) were sacrificed by decapitation without anesthesia and both sciatic nerves were isolated. The nerves were transferred to a Petri dish and maintained for ~15 min in ice-cold high-Mg^2+^ ACSF containing in mM: 124 NaCl, 1.25 NaH_2_PO_4_, 10 MgSO_4_, 2.7 KCl, 26 NaHCO_3_, 2 CaCl_2_, 2 ascorbic acid, 18.6 glucose. The nerves were fixed for 1 h in 4% paraformaldehyde (PFA) prepared in phosphate-buffered saline (PBS). PBS contained, in mM: 4.3 Na_2_HPO_4_, 1.6 NaH_2_PO_4_, 150 NaCl. Afterwards the nerves were washed with PBS (3 times × 15 min) and transferred to 30% sucrose solution in PBS, where they were kept overnight at 4°C. The nerves were then embedded into Tissue-Tek (Sakura Finetek Europe, Netherlands) and frozen at −80°C. Ten micrometre thick slices were prepared with a Leica CM3050S Cryotome and Leica 819 Microtome blades, and transferred onto the glass slides. The slices were washed (3 times × 15 min) with tris-buffered saline (TBS), and incubated in blocking solution for 2 h at room temperature. TBS contained, in mM: 100 Sigma 7–9, 154 NaCl. Blocking solution contained: 3% bovine serum albumin and 0.2% Triton-X in TBS. The slices were incubated with primary antibody overnight at 4°C, washed in TBS (3 times × 15 min), and incubated with secondary antibody coupled to a fluorescent dye for 3–4 h at room temperature. For double and triple immune-labeling the antibodies were applied sequentially, i.e., first primary followed by first secondary, followed by second primary, followed by second secondary, etc. All antibodies were applied in the blocking solution. Washing of slices with TBS (3 times × 15 min) was performed after incubation with each antibody. At the end of the immuno-labeling procedure counterstain 4′,6-diamidino-2-phenylindole (DAPI, 5 mg/ml) was applied for 5 min at room temperature. The slices were washed with water, dried, covered with Vectashield (Vector Laboratories, Inc, Burlingame, CA, USA), and sealed with nail-polish. The list of antibodies used in this study is given in Table 1. Confocal images were acquired with confocal laser scanning microscope LSM-710 (Zeiss, Germany) equipped with 40× objective (Plan-Apochromat 40×/1.3 Oil DIC M27, Zeiss, Germany). The dyes were excited with the following laser-lines: 405 nm for DAPI, 488 nm for Alexa-Fluor-488, 568 nm for rhodamine-red-X (RRX) or Cy3, and 633 nm for Alexa-Fluor-633 or Cy5. The pinhole was set to 34–38 μm depending on the wave-length, and was adjusted so that the optical section for each channel was 0.9 μm. Images for multiple channels were acquired sequentially, and care was taken that parts of the emission spectra from which the light was collected for different dyes do not overlap. Images were further analyzed with ZEN Software (Zeiss, Germany).

**Table 1:** **List of antibodies used for immunohistochemistry**.

Antibody name	Antibody number and the company name	Dilution
**Primary antibodies**
Rabbit anti-Ca_v_1.2 (L-type VGCCs)	AB5156, Millipore	1:100
Rabbit anti-CACNA 1B	ab66426, Abcam	1:100
(N-type VGCCs)
Goat anti-choline	AB144P, Millipore	1:100
acetyltransferase (ChAT)
Rat anti-myelin basic protein (MBP)	ab7349, Abcam	1:125
Chicken anti-neurofilament 200 kDa	ab4680, Abcam	1:1000
**Secondary antibodies**
Goat anti-chicken Alexa-Fluor-488	A-11039, Invitrogen	1:500
Goat anti-rabbit Rhodamine-Red-X	111-295-003, Dianova	1:500
Goat anti-rat Alexa-Fluor-633	A-21094, Invitrogen	1:500
Donkey anti-rabbit Alexa-Fluor-488	A-21206, Invitrogen	1:500
Donkey anti-goat-Cy3	705-165-003, Dianova	1:500
Donkey anti-chicken-Cy5	703-176-155, Dianova	1:500

### Chemicals and Drugs

All chemicals were obtained from Sigma (Taufkirchen, Germany) or Carl Roth (Karlsruhe, Germany). OGB-1, Magnesium Green, and pluronic acid were obtained from Invitrogen (LifeTechnologies GmbH, Darmstadt, Germany). Tetrodotoxin (TTX), TTA-P2, ω-conotoxin GVIA, and ω-agatoxin IVa were obtained from Alomone Labs (Jerusalem, Israel). Stock solutions were prepared according to manufacturer’s instructions and stored at −20°C.

### Statistics

Statistical analysis was performed using SPSS statistics Software (Version 23.0, IBM Corp. Armonk, NY, USA). Statistical significance of the drug effect was determined with paired-samples *T*-test. All values are shown as the mean ± SEM. Differences were considered significant at *p* < 0.05 (*p < 0.05, **p < 0.01, ***p < 0.001).

## Results

### Electrical Stimulation of Nerve Bundles Triggers Ca^2+^ Transients Along Sciatic Nerve Axons

**Figure 1:**
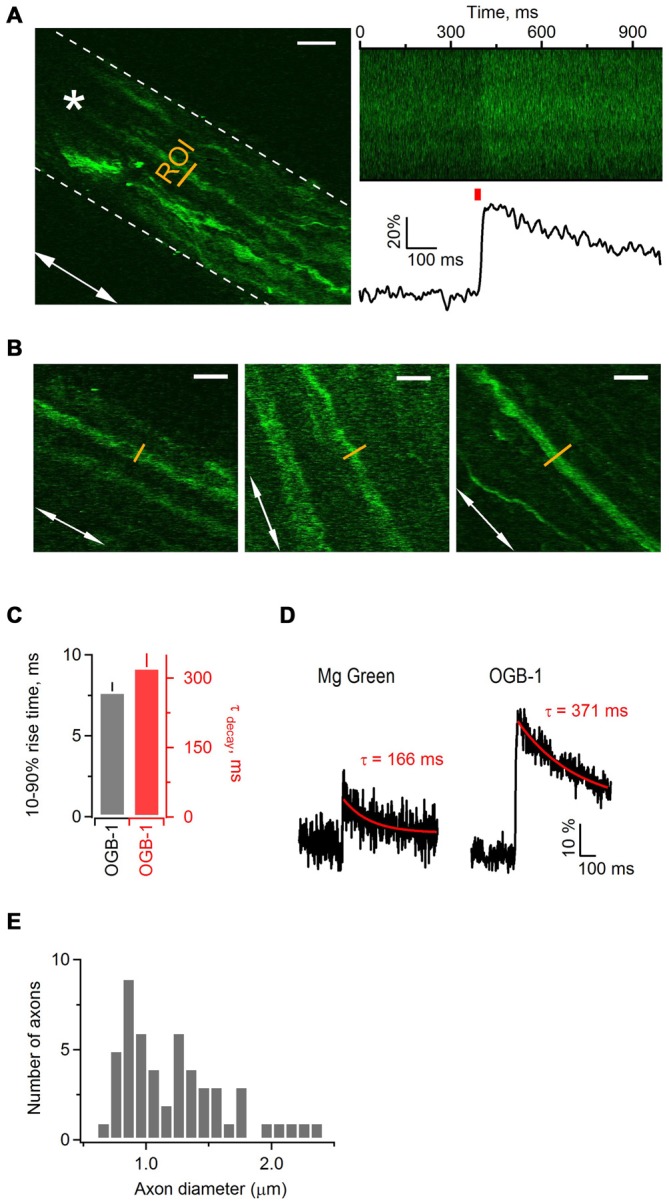
**Electrical stimulation triggers Ca^2+^ transients along axonal shafts in neonatal mouse sciatic nerve. (A)** *Left*: 2-photon image (single plane) of an axonal bundle filled with Ca^2+^ indicator Oregon Green BAPTA-1 (OGB-1) in a mouse sciatic nerve slice. The white dotted line delineates the contour of the axonal bundle. The white arrow shows orientation of the axons in the bundle. The yellow line indicates the ROI located ~300 μm away from the injection site. The white star indicates the position of stimulation electrode. Scale bar 20 μm. **(A)** *Right, top*: plot of fluorescence intensity in the ROI, indicated on the left, vs. time. The plot was obtained during the line scan along the ROI (500 lines scanned at 500 Hz). **(A)** *Right, bottom*: corresponding example trace showing increase in fluorescence intensity of OGB-1 upon electrical stimulation of axonal bundle with a single pulse. The trace is an average of three successive sweeps. The red bar indicates the time point of electrical stimulation. **(B)** Examples of imaged axons included in the study. White arrows show the orientation of axons in each neuronal bundle. The yellow line indicates the region of interest ROI located ~300 μm away from the injection site. Scale bars 12 μm. **(C)** Bar-graphs showing average 10–90% rise time of 7.73 ± 0.56 ms (mean ± SEM; *n* = 6) and average decay time constant of 323 ± 30 ms (mean ± SEM; *n* = 7) for Ca^2+^ transients recorded with OGB-1. **(D)** Example traces showing increase in fluorescence intensity of Magnesium Green (left) or OGB-1 (right) upon electrical stimulation of sciatic nerve axons with a single pulse. Each trace is an average of three successive sweeps. Red lines show mono-exponential fit to the decaying phase of each transient; corresponding decay time constants (tau) are indicated above each transient. **(E)** Histogram showing the diameter size distribution of the thin axons within bundles. Fifty-two axons within 18 bundles were analyzed.

The first goal was to test whether activity-dependent Ca^2+^ transients occur along mouse sciatic nerve axons in a millisecond time domain, and to assess whether high- or low-affinity indicator works best to measure these transients. We performed 2-photon Ca^2+^ imaging in nerve slices filled with a high-affinity Ca^2+^ indicator OGB-1 AM (K_d_ = 170 nM) or a low-affinity Ca^2+^ indicator Magnesium Green (K_d_ = 6 μM), while stimulating axons electrically (Figures 1A,D). We aimed to image small axonal bundles which had constant diameter (in the range of 3–12 μm) over the length of tens of micrometers (Figure 1B). We estimated that the diameter of thin axons comprising these bundles was in the range of 0.6–2.4 μm (Figure 1E). Each region of interest (ROI) was selected as a line placed perpendicular to the orientation of the axons (Figure 1A). We avoided to image cellular structures appearing as varicosities and potentially being growth cones or cut-and-resealed axons. To ensure that we record Ca^2+^ transients selectively in axons, but not in the developing Schwann cells, we acquired all scans far from the indicator injection site (>300 μm). This was important, as we observed that at the injected site both Schwann cells and axons took up the dye, while far from the injection site only axons were stained with the indicator and no glial cells were labeled (Figure 1A 1A left,B). Based on the previous studies (Thaxton et al., 2011) and our own unpublished observations, the end-to-end length of a Schwann cell in the sciatic nerve slice prepared from a neonatal mouse is no longer than 300 μm. In addition, Schwann cells in neonatal sciatic nerve are not coupled via gap-junctions (own unpublished observation). Hence, at the distance of >300 μm from the injection site, which exceeds the length of a Schwann cell in our preparation, we could selectively image the axons.

While stimulating sciatic nerve axons electrically with single pulses every 30 s, we repeatedly executed fast line-scans (500 Hz) perpendicular to the orientation of the axons, and tracked changes in the fluorescence of OGB-1 (Figure 1A right) or Magnesium Green (not shown). Stimulation of axons led to a fast increase of Ca^2+^-dye fluorescence which then decayed back to baseline, indicating that changes in Ca^2+^ level occur in sciatic nerve axons upon electrical activity (Figure 1A right). The peak amplitude of Ca^2+^ transients in axons loaded with OGB-1 and stimulated with single pulse was typically several times larger than in axons loaded with Magnesium Green, and the signal-to-noise ratio was much better with OGB-1 compared to Magnesium Green (Figure 1D). Further, with Magnesium Green we usually had to stimulate the axons with trains (e.g., 3–50 pulses at 25–100 Hz) rather than with single pulses in order to detect Ca^2+^ transients. The 10–90% rise-time and the decay time constant of Ca^2+^ transients recorded with OGB-1 were 7.73 ± 0.56 ms (*n* = 6) and 323 ± 30 ms (*n* = 7), respectively (Figure 1C). Ca^2+^ transients recorded with Magnesium Green were very small upon single pulse stimulation, therefore it was difficult to estimate rise and decay time reliably even when several sweeps were averaged. We could do it only in one experiment where the 10–90% rise-time was 4.48 ms and the decay time constant was 166 ms (Figure 1D). Based on these findings we decided to use a high-affinity Ca^2+^ indicator OGB-1 for our experiments, aiming for higher signal sensitivity but keeping in mind that OGB-1 likely reports an overestimate of rise- and decay time of Ca^2+^ transients along the axons (Regehr, 2000).

### Ca^2+^ Transients Along Sciatic Nerve Axons Depend on TTX-Sensitive Action Potentials

**Figure 2:**
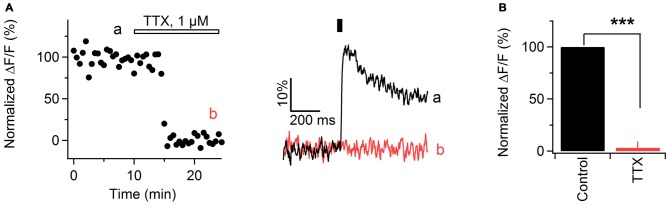
**Ca^2+^ transients in mouse sciatic nerve depend on action potential propagation. (A)** *Left*: time course of the amplitude of axonal Ca^2+^ transients evoked by single pulse stimulation, before and during bath application of 1 μM tetrodotoxin (TTX). “a” and “b” indicate time-period from which the 10 successive sweeps before and after TTX application were averaged (corresponding averages are shown on the right). Horizontal bar indicates time-period of TTX application. Ca^2+^ transients are recorded in axons filled with OGB-1. **(A)** *Right*: examples traces recorded before (black) and during (red) TTX application. Each example trace represents an average of 10 successive sweeps. Black vertical bar indicates time-point of electrical stimulation. **(B)** Summary bar graphs showing the effect of TTX on the amplitude of Ca^2+^ transients vs. control. The TTX application reduces the fluorescence by 97 ± 6% (mean ± SEM) of control values (*n* = 6; ***p < 0.001).

In brain slices, electrical stimulation of gray and white matter axons results in activation of VGCCs located in presynaptic boutons or along axonal shafts (Koester and Sakmann, 2000; Kukley et al., 2007). This activation depends on action potentials mediated by TTX-sensitive Na^+^ channels. As peripheral nerves contain both TTX-sensitive and TTX-resistant Na^+^ channels (Kostyuk et al., 1981), we tested whether Ca^2+^ transients in sciatic nerve axons are inhibited by TTX. We stimulated the axons electrically with single pulses at 0.033 Hz and acquired line-scans as described above. After verifying that the amplitude of evoked Ca^2+^ transients remains stable for at least 10 min, we applied TTX (1 μM) via the bath. TTX reduced the peak amplitude of Ca^2+^ transients by 97 ± 6% (Figures 2A,B) indicating that Ca^2+^ transients along the axons depend on action potentials mediated by TTX-sensitive Na^+^ channels. However, in one experiment we found that the amplitude of Ca^2+^ transients was decreased only by 68% upon TTX application (not shown), suggesting that TTX-resistant Na^+^ channels and/or Na^+^-action-potential independent mechanisms may partially mediate evoked Ca^2+^ increase along sciatic nerve axons.

### Ca^2+^ Transients Along Sciatic Nerve Axons Involve Ca^2+^ Influx from the Extracellular Space

**Figure 3:**
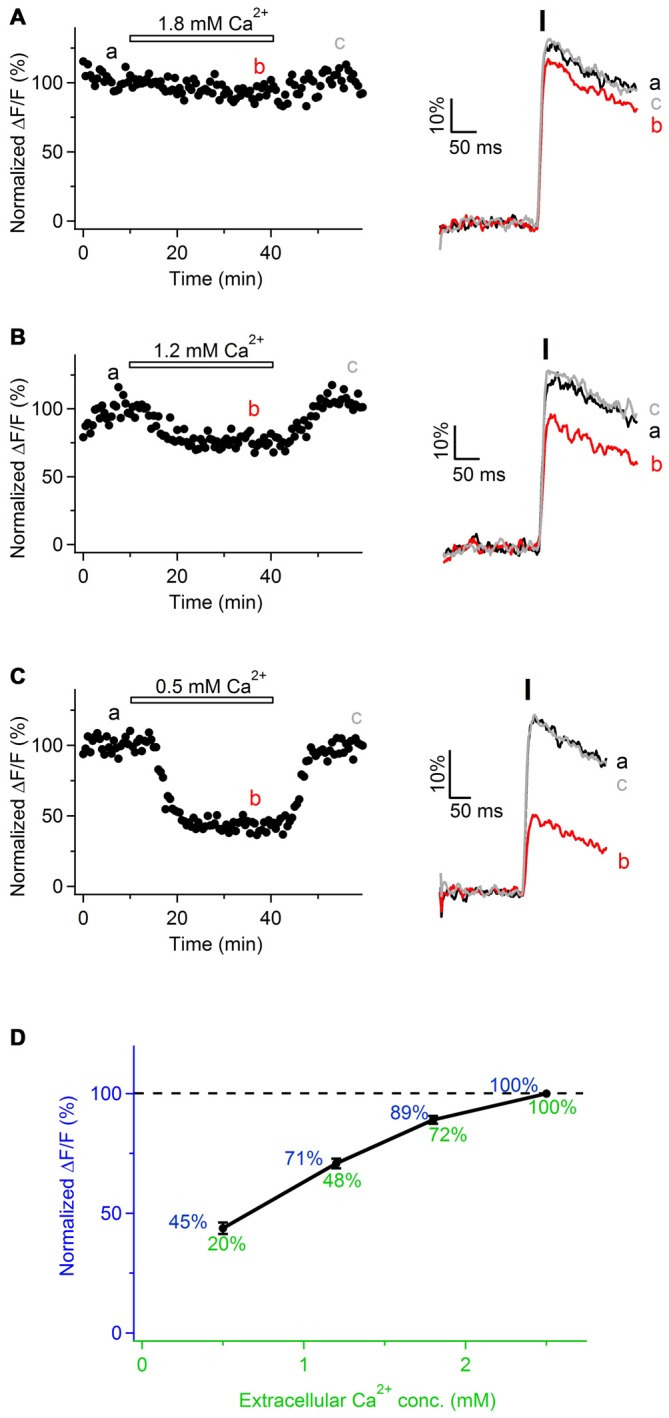
**Ca^2+^ transients in mouse sciatic nerve involve Ca^2+^ influx from the extracellular space. (A–C)** *Left*: time course of the amplitudes of axonal Ca^2+^ transients evoked by single pulse stimulation, before, during and after bath application of extracellular solution containing reduced Ca^2+^ concentration: 1.8 mM (**A**, *n* = 3), 1.2 mM (**B**, *n* = 3), and 0.5 mM (**C**, *n* = 3). Ca^2+^ concentration in the control solution was 2.5 mM. Horizontal bars indicate time-period when solution with reduced Ca^2+^ concentration was applied. Amplitude of Ca^2+^ transients is normalized on the amplitude of the transients obtained in the presence of 2.5 mM external Ca^2+^. “a”, “b”, and “c” indicate time-period from which the 10 successive sweeps were averaged (corresponding averages are shown on the right of each time course). Ca^2+^ transients are recorded in axons filled with OGB-1. **(A–C)** *Right*: example traces recorded before (black), during (red), and after (gray) bath application of extracellular solution with reduced Ca^2+^ concentration. Each example trace represents an average of 10 successive sweeps. Black vertical bar indicates time-point of electrical stimulation. **(D)** Summary graph showing dependence of the normalized amplitude of Ca^2+^ transients (ΔF/F, blue) on Ca^2+^ concentration in the extracellular solution (green). Values of the normalized amplitude of Ca^2+^ transients are shown in blue above the curve. Values of extracellular Ca^2+^ concentration normalized to 2.5 mM are shown in green below the curve.

To investigate the origin of Ca^2+^ transients in peripheral nerve axons, we perfused the slices with ACSF containing reduced Ca^2+^ concentration (1.8, 1.2 or 0.5 mM Ca^2+^ instead of 2.5 mM). The total divalent concentration was maintained constant by adjusting the levels of Mg^2+^ ions in the bath. Under these conditions, the peak amplitude of Ca^2+^ transients was reversibly reduced by 11 ± 2% in 1.8 mM Ca^2+^ (Figures 3A,D; *n* = 3), 29 ± 3% in 1.2 mM Ca^2+^ (Figures 3B,D; *n* = 3), and 55 ± 3% in 0.5 mM Ca^2+^ (Figures 3C,D; *n* = 3). These findings suggest that evoked Ca^2+^ transients along sciatic nerve axons involve Ca^2+^ influx from the extracellular space.

Notably, in the experiments with various Ca^2+^ concentration in the bath we observed that relationship between the peak amplitude of Ca^2+^ transients (ΔF/F) and extracellular Ca^2+^ concentration is not linear, but Ca^2+^ influx tends to saturate with increasing Ca^2+^ level in the bath (Figure 3D). This non-linearity may be explained by the fact that Ca^2+^-binding site(s) at the membrane surface or within the channel pore, to which Ca^2+^ ions have to bind in order to pass through the channel, get saturated at higher extracellular Ca^2+^ concentration (Augustine and Charlton, 1986; Mintz et al., 1995). Alternatively, the observed non-linearity may be explained by slight saturation of OGB-1 when Ca^2+^ transients are recorded with 2.5 mM Ca^2+^ in the bath.

### Ca^2+^ Transients Along Sciatic Nerve Axons are Mediated By VGCCs

**Figure 4:**
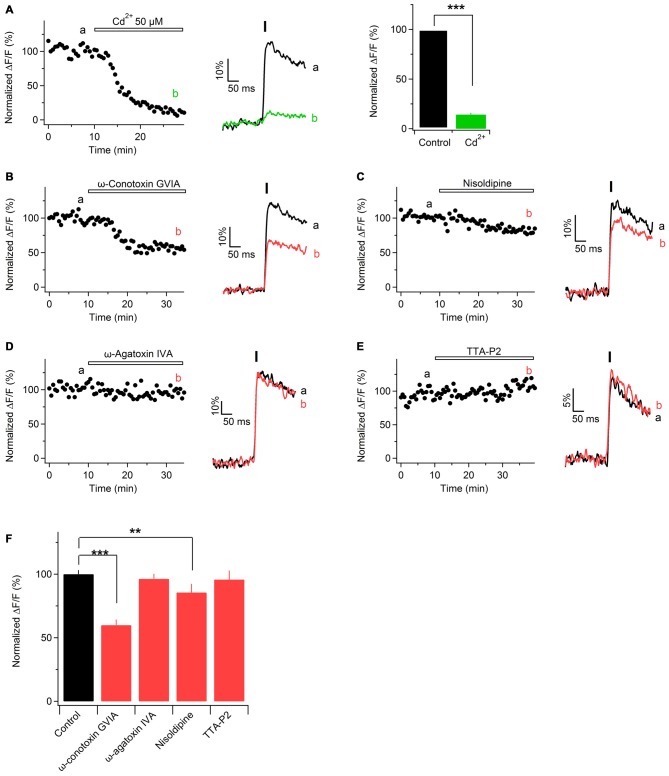
**Ca^2+^ transients in mouse sciatic nerve are mediated by voltage-gated Ca^2+^ channels (VGCCs). (A)** *Left*: time course of the amplitudes of axonal Ca^2+^ transients evoked by single pulse stimulation before and during application of Cd^2+^. “a” and “b” indicate the time-period from which 10 successive sweeps were averaged (corresponding average is shown on the right of the time course). Horizontal bar indicates time-period of Cd^2+^ application. **(A)** *Middle*: example traces recorded before (black) and during (green) application of 50 μM Cd^2+^. Each example trace represents an average of 10 successive sweeps. Black vertical bar indicates time-point of electrical stimulation. **(A)** *Right*: summary bar graphs showing the effect of Cd^2+^ on the amplitude of Ca^2+^ transients vs. control. Fifty micromolar Cd^2+^ reduces the fluorescence by 86 ± 2% (mean ± SEM) of control values (*n* = 6; ***p < 0.001). **(B–E)** *Left*: time course of the amplitudes of axonal Ca^2+^ transients evoked by single pulse stimulation, before and after application of a specific blocker of voltage-gated Ca^2+^ channels, VGCCs: N-type VGCCs blocker ω-conotoxin GVIA, 1 μM (**B**, *n* = 4); L-type VGCCs blocker nisoldipine, 1 μM (**C**, *n* = 5); P/Q-type VGCCs blocker ω-agatoxin IVA, 500 nM (**D**, *n* = 4), and T-type VGCCs blocker TTA-P2, 1 μM (**E**, *n* = 5). “a” and “b” indicate the time-period from which 10 successive sweeps were averaged (corresponding average is shown on the right of the time course). Horizontal bar indicates time-period of VGCCs blocker application. **(B–E)** *Right*: example traces recorded before (black) or during (red) bath application of a VGCCs blocker. Each example trace represents an average of 10 successive sweeps. Black vertical bar indicates time-point of electrical stimulation. **(F)** Summary bar graphs showing the effect of specific VGCCs blockers on the amplitude of Ca^2+^ transients vs. control. The amplitude was reduced in the presence of N-type VGCC blocker ω-conotoxin GVIA (1 μM, *n* = 4, ***p < 0.001), as well as in the presence of L-type VGCC blocker nisoldipine (1 μM, *n* = 5, **p < 0.01). On the contrary, the amplitude was unaffected by the P/Q- and T-type VGCCs blockers, ω-agatoxin IVA (500 nM, *n* = 4) and TTA-P2 (1 μM, *n* = 5), respectively.

In physiological or pathological conditions Ca^2+^ transients along the axons can be mediated by Ca^2+^ entry through different routes, e.g., VGCCs (Lev-Ram and Grinvald, 1987; Fern et al., 1995; Sun and Chiu, 1999; Brown et al., 2001; Zhang et al., 2006; Alix et al., 2008; Sargoy et al., 2014), reversal of Na^+^/Ca^2+^ exchanger (Lehning et al., 1996), release from intracellular stores (Matute, 2010; Villegas et al., 2014), or ionotropic Ca^2+^-permeable receptor channels (Kinkelin et al., 2000; Matute, 2010). Our goal was to test whether VGCCs contribute to Ca^2+^ entry along peripheral nerve axons. To address this question, we stimulated sciatic nerve axons electrically, acquired fast line-scans and perfused the slices with ACSF containing Cd^2+^ (50 μM), a broad-spectrum VGCCs blocker. Cd^2+^ strongly decreased the peak amplitude of Ca^2+^ transients, i.e., by 86 ± 2% (Figure 4A; *n* = 6). We also tested whether application of higher concentration of Cd^2+^, i.e., 100 μM, results in a stronger inhibition of evoked Ca^2+^ transients. This was indeed the case, as Ca^2+^ transients were inhibited by 94 ± 3% *p* < 0.01 (not shown; *n* = 3). However, application of 100 μM Cd^2+^ also caused a steady increase of both basal and peak fluorescence of OGB-1. Increase of Ca^2+^ indicator fluorescence in the presence of Cd^2+^ has also been reported in other preparations, e.g., in cultured hippocampal neurons (Ermolyuk et al., 2013) or in rat vagus nerve fibers (Wächtler et al., 1998). Possible explanations of this phenomenon are: rise of intracellular Ca^2+^ levels triggered by Cd^2+^ (Ermolyuk et al., 2013), binding of Cd^2+^ ions to the Ca^2+^ indicator (Regehr and Atluri, 1995) or block of Ca^2+^-ATPase by Cd^2+^ with subsequent accumulation of intracellular Ca^2+^ (Yuan et al., 2013). Taken together, our findings suggest that VGCCs contribute to Ca^2+^ transients along sciatic nerve axons.

### Pharmacological Characterization of VGCCs Located Along Sciatic Nerve Axons

According to their electrophysiological and pharmacological properties VGCCs are classified into L-, N-, P/Q-, R-, and T-type (Dolphin, 2006). L-, N-, P/Q-, and R-type channels (also called high-voltage activated channels) open to large membrane depolarization and show a long-lasting current (Bean, 1985). T-type channels (also called low-voltage activated channels) open by smaller voltage changes and show a transient current (Huguenard, 1996). To investigate which subtypes of VGCCs are involved in Ca^2+^ entry along peripheral nerve axons, we challenged evoked Ca^2+^ transients with specific blockers of different VGCCs subtypes. Application of N-type VGCCs blocker ω-conotoxin GVIA (1 μM) reduced the peak amplitude of Ca^2+^ transients by 40 ± 3% (Figures 4B,F; *n* = 4). L-type VGCCs blockers nisoldipine (1 μM) or nifedipine (10 μM) inhibited the peak amplitude of Ca^2+^ transients by 15 ± 3% (Figures 4C,F; *n* = 5) or by 12 ± 7% *p* = 0.32 (not shown; *n* = 2), respectively. In contrast, application of P/Q-type VGCCs blocker ω-agatoxin IVa (500 nM) or T-type VGCCs blocker TTA-P2 (1 μM) did not significantly change the peak amplitude of Ca^2+^ transients: amplitude in the presence of ω-agatoxin IVa was reduced by 4 ± 6% (Figures 4D,F; *n* = 4) while in the presence of TTA-P2 it was reduced by 4.2 ± 7% (Figures 4E,F; *n* = 5). These results indicate that Ca^2+^ influx along sciatic nerve axons is partially mediated by N- and L-type VGCCs while P/Q and T-type VGCCs are not involved.

### Immunohistological Evidence for VGCCs in the Mouse Sciatic Nerve

**Figure 5:**
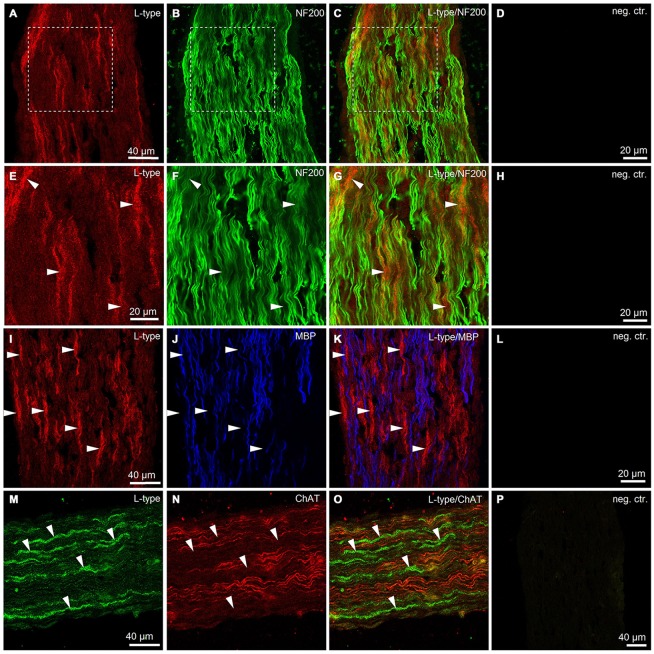
**Immunohistological evidence for L-type VGCCs in the developing mouse sciatic nerve. (A)** Confocal image (single plane) showing labeling of sciatic nerve axons with antibody against L-type VGCC (red, rhodamine-red-X, RRX). **(B)** Confocal image (single plane) showing labeling of sciatic nerve axons with antibody against neurofilament 200 kDa, NF200 (green, Alexa-Fluor-488). **(C)** Overlay of green and red channels. **(D)** Negative control, i.e., both primary antibodies are omitted. Scale bar shown in **(A)** is the same for images **(A–C)**. Dotted white boxes in panels **(A–C)** indicate the region for which the higher magnification images are shown in panels **(E–G)**. **(E–G)** Higher magnification images of the region marked with white boxes in panels **(A–C)**. Note that mainly the thin axons labeled weakly with neurofilament 200 kD are stained for L-type VGCCs (arrowheads). **(H)** Negative control, i.e., both primary antibodies are omitted. Scale bar shown in **(E)** is the same for images **(E–G)**. **(I)** Confocal image (single plane) showing labeling of sciatic nerve axons with antibody against L-type VGCCs (red, RRX). **(J)** Confocal image (single plane) showing labeling of sciatic nerve axons with antibody against myelin basic protein, MBP (blue, Alexa-Fluo-633). **(K)** Overlay of red and blue channels. **(L)** Negative control, i.e., both primary antibodies are omitted. Scale bar shown in **(I)** is the same for images **(I–K)**. Note absence of co-localization between L-type VGCCs and MBP (arrowheads). **(M)** Confocal image (single plane) showing labeling of sciatic nerve axons with antibody against L-type VGCCs (green, Alexa-Fluor-488). **(N)** Confocal image (single plane) showing labeling of sciatic nerve axons with antibody against choline acetyltransferase, ChAT (red, Cy3). **(O)** Overlay of green and red channels. **(P)** Negative control, i.e., both primary antibodies are omitted. Scale bar shown in **(M)** is the same for images **(M–O)**. Note absence of co-localization between L-type VGCCs and ChAT (arrowheads). The example images shown in **(A–H)**, **(I–L)**, and **(M–P)** are from three different animals, respectively.

**Figure 6:**
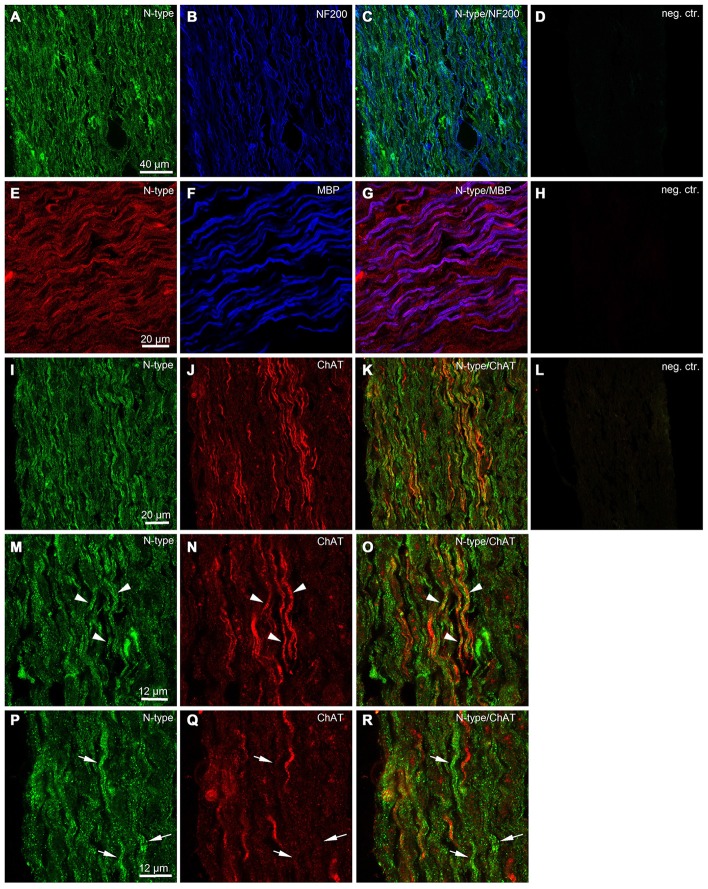
**Immunohistological evidence for N-type VGCCs in the developing mouse sciatic nerve. (A)** Confocal image (single plane) showing labeling of sciatic nerve axons with antibody against N-type VGCC (green, Alexa-Fluor-488). **(B)** Confocal image (single plane) showing labeling of sciatic nerve axons with antibody against neurofilament 200 kDa (blue, Cy5). **(C)** Overlay of green and blue channels. **(D)** Negative control, i.e., both primary antibodies are omitted. Scale bar shown in **(A)** is the same for images **(A–D)**. **(E)** Confocal image (single plane) showing labeling of sciatic nerve axons with antibody against N-type VGCCs (red, RRX). **(F)** Confocal image (single plane) showing labeling of sciatic nerve axons with antibody against (MBP) blue, Alexa-Fluor-633. **(G)** Overlay of red and blue channels. **(H)** Negative control, i.e., both primary antibodies are omitted. Scale bar shown in **(E)** is the same for images **(E–H)**. **(I)** Confocal image (single plane) showing labeling of sciatic nerve axons with antibody against N-type VGCCs (green, Alexa-Fluor-488). **(J)** Confocal image (single plane) showing labeling of sciatic nerve axons with antibody against ChAT (red, Cy3). **(K)** Overlay of green and red channels. **(L)** Negative control, i.e., both primary antibodies are omitted. Scale bar shown in **(I)** is the same for images **(I–L)**. **(M)** Confocal image (single plane) showing labeling of sciatic nerve axons with antibody against N-type VGCCs (green, Alexa-Fluor-488). **(N)** Confocal image (single plane) showing labeling of sciatic nerve axons with antibody against ChAT (red, Cy3). **(O)** Overlay of green and red channels. Arrowheads indicate axons co-labeled with N-type VGCCs and ChAT. Scale bar shown in **(M)** is the same for images **(M–O)**. **(P)** Confocal image (single plane) showing labeling of sciatic nerve axons with antibody against N-type VGCCs (green, Alexa-Fluor-488). **(Q)** Confocal image (single plane) showing labeling of sciatic nerve axons with antibody against ChAT (red, Cy3). **(R)** Overlay of green and red channels. Arrows indicate axons labeled with N-type VGCCs but negative for ChAT. Scale bar shown in **(P)** is the same for images **(P–R)**. The example images shown in **(A–D)** and **(I–R)** are from the same animal, while the example images shown in **(E–H)** are from another animal.

To obtain additional independent evidence for the presence of VGCCs in the developing mouse sciatic nerve, we performed immunohistochemistry. We found that both L- and N-type VGCCs were present in the nerve, but their localization was different. L-type VGCCs appeared on bundles of thin axons which often showed weaker labeling with neurofilament (NF200) than the other axons in the nerve (*n* = 3 animals, Figures 5A–H). The axons expressing L-type VGCCs showed no co-labeling with myelin basic protein (MBP; *n* = 3 animals, Figures 5I–L) or choline acetyltransferase (ChAT), a marker of motor axons (*n* = 3 animals, Figures 5M–P). These findings suggest that L-type VGCCs are expressed by non-myelinated sensory fibers. N-type VGCCs appeared on myelinated axons (*n* = 3 animals, Figures 6E–H) which were also positive for NF200 (*n* = 3 animals, Figures 6A–D). Yet the resolution of our confocal system did not allow to reliably conclude whether N-type VGCCs were expressed solely on the axonal membrane or on the myelin as well. Some axons positive for N-type VGCCs co-labeled with ChAT (*n* = 3 animals, Figures 6I–O), while other axons expressing N-type VGCCs were negative for ChAT (*n* = 3 animals, Figures 6I–L,P–R). These data point to the fact that N-type VGCCs are present on myelinated sensory and motor fibers.

## Discussion

The first important finding of the present study is that transient increases in axoplasmic Ca^2+^ concentration take place in axonal shafts of neonatal mouse peripheral nerve when axons are stimulated electrically with single pulses. Further, we show for the first time that Ca^2+^ transients in peripheral nerves *in situ* are fast, i.e., occur in a millisecond time-domain. Up to now few studies have reported transient activity-dependent Ca^2+^ elevations along peripheral nerve axons *in situ* (Elliott et al., 1989; Quasthoff et al., 1995, 1996; Wächtler et al., 1998; Mayer et al., 1999; Jackson et al., 2001). However, no reasonable conclusion regarding kinetic parameters of Ca^2+^ transients can be made from these studies because the time-course of Ca^2+^ transients is rate-limited by slow acquisition, i.e., slow frame scanning mode and low sampling rate (~2.5 Hz; Jackson et al., 2001). At the same time Ca^2+^ transients with fast kinetics have been reported in axons of dorsal root ganglion neurons in culture but the involvement of VGCCs in Ca^2+^ elevations in culture has not been investigated (Lüscher et al., 1996). In the present study we used fast acquisition mode, i.e., line-scanning at 500 Hz, and found that action potentials in mouse sciatic nerve axons *in situ* trigger axoplasmic Ca^2+^ elevations which rise relatively fast (10–90% rise-time is ~7.7 ms) and decay back to baseline with a slower time constant τ of ~320 ms, as estimated with high-affinity Ca^2+^ indicator OGB-1. These values are quite similar to those obtained with a Ca^2+^ indicator of comparable K_d_ in other preparations, including mouse cerebellar mossy fiber boutons (Delvendahl et al., 2015) and presynaptic terminals of rat calyx of Held (Borst et al., 1995). Remarkably, aiming for sufficient sensitivity and good signal-to-noise ratio during imaging of small axons in neonatal mouse nerve, we selected a high-affinity Ca^2+^ indicator OGB-1 (K_d_ = 170 nM) for our experiments. The shortcoming of this experimental design is that OGB-1 may be too slow to precisely follow rapid changes in intra-axonal Ca^2+^ concentration, and most likely also adds some buffer capacity to the axoplasm (Regehr and Atluri, 1995). Hence, the factual activity-dependent Ca^2+^ dynamics in the axoplasm is likely to be even faster than reported by OGB-1. Taken together, our findings indicate that transient activity-dependent Ca^2+^ elevations along peripheral nerve axons can occur on a rapid time-scale, similar as it happens at synaptic boutons or along axonal shafts in the CNS.

The second important finding of our study is that activity-dependent Ca^2+^ transients along peripheral nerve axons in neonatal mouse depend on Ca^2+^ influx from extracellular space and involve activation of N- and L-type VGCCs. We found that a blocker of N-type VGCCs, ω-conotoxin GVIA, reduced the amplitude of Ca^2+^ transients by ~40%, the blockers of L-type VGCCs nisoldipine or nifedipine caused ~15% reduction, while the blockers of P/Q- and T-type channels were ineffective. Furthermore, the results of our immunohistological experiments suggest that in the developing mouse sciatic nerve L-type VGCCs are present on non-myelinated sensory fibers, while N-type channels appear on myelinated motor and sensory axons. To the best of our knowledge, this is the first report about subtypes of functional VGCCs present along the peripheral nerve axons in neonatal mice. Interestingly, in neonatal rodent central (optic) nerve likewise L- or N-type VGCCs were suggested to be of functional significance (Sun and Chiu, 1999; Alix et al., 2008), while P/Q-type channels seem to get involved later during development (Alix et al., 2008). L- and/or N-type VGCCs also mediate Ca^2+^ influx in adult optic nerve during pathological conditions (Fern et al., 1995; Brown et al., 2001). When we compared our findings on VGCCs subtypes in neonatal mouse sciatic nerve to another preparation of peripheral nerve, i.e., adult mouse postganglionic sympathetic axon bundle, it turned out that also in those axons ~40% of the total Ca^2+^ influx is carried by N-type VGCCs, however, in contrast to our findings, L-type VGCCs were not involved (Jackson et al., 2001). In adult mouse C-fibers T-type VGCCs have been suggested to play a role in modulating action potential conduction velocity (François et al., 2015), however in the neonatal mouse we could not find T-type VGCCs contribution to Ca^2+^ influx along the axons. Remarkably, at the mammalian neuromuscular junction, where some of the peripheral nerve axons terminate, P/Q-type represents the major subtype of VGCCs, although L- and N-type VGCCs also play a role during development, re-innervation or pathological conditions (Katz et al., 1996; Nudler et al., 2003). At the distal nerve endings, in turn, T-type VGCCs have been found in addition to other VGCCs subtypes (François et al., 2015). Hence the specific subtypes of VGCCs are likely targeted differently to different functional compartments of the same axon, and may be also differently regulated in developing and adult animals, as well as during pathological conditions. In addition to known VGCCs subtypes other yet unidentified subtypes of VGCCs, or alternative routes (e.g., reversed Na^+^/Ca^2+^ exchanger, release from internal store), may contribute to activity-dependent Ca^2+^ entry into the axoplasm of peripheral nerve axons. In line with this idea are our findings that Ca^2+^ transients in mouse sciatic nerve are reduced only partially by specific blockers of VGCCs. Furthermore, also in the unmyelinated nerve fibers of rat vagus nerve neither L- nor N-type nor P/Q-type VGCCs mediated Ca^2+^ entry along the axons, although Ca^2+^ transients in that preparation were largely inhibited by Cd^2+^ (Wächtler et al., 1998). Hence, more experiments in various preparations of central and peripheral nerves/white matter are required to clarify this issue.

Why do peripheral nerve axons express VGCCs along their shafts, and what could be the functional significance of activity-dependent axonal Ca^2+^ transients under physiological circumstances? Ca^2+^ is involved in the majority of cellular functions. Importantly, as cells keep free cytosolic Ca^2+^ level very low (~100 nM), what determines the specificity and the functional output of each Ca^2+^-dependent process is the amplitude, the time-course and the spatial domain of a transient change in intracellular Ca^2+^ concentration (Berridge et al., 2003). Fast (microseconds to milliseconds) Ca^2+^ transients are usually involved in fast cellular processes, e.g., synaptic transmission, opening of Ca^2+^-dependent channels, muscle contraction, etc (Berridge et al., 2003). At axonal synaptic terminals, for example, highly localized (nano- or microdomains) rapidly rising (<1 ms) and large (~20 fold) Ca^2+^ elevations mediated by VGCCs trigger rapid release of synaptic vesicles and ensure high precision of synaptic signaling (Kandel et al., 2000). In turn, more global residual Ca^2+^ changes, which are also slower and smaller in amplitude, contribute to modulation of transmitter release, e.g., synaptic potentiation (Swandulla et al., 1991; Wang and Augustine, 2015). We want to emphasize that as Ca^2+^ transients recorded along peripheral nerve axons in our study rise in a range of few milliseconds (10–90% rise time ~7.7 ms) and this time probably underestimates the true speed of Ca^2+^ influx into the axon upon action potential propagation, these Ca^2+^ transients are well suited to trigger or/and modulate relatively fast axonal processes. For example, transient increase in axoplasmic Ca^2+^ concentration may be involved in regulation of action potential conduction or frequency through e.g., activation of Ca^2+^-dependent K^+^ and/or Cl^−^ channels, or inactivation of Ca^2+^ channels (Jirounek et al., 1991; Lüscher et al., 1996; Sun and Chiu, 1999; Alix et al., 2008). Another possible function of VGCCs and fast Ca^2+^ entry in peripheral axons, rarely considered in the literature, could be the contribution to neurotransmitter release (vesicular or non-vesicular) along axonal shafts. Rapid (few milliseconds) increases in Ca^2+^ concentration mediated by VGCCs take place along axonal shafts in white matter of the CNS (Lev-Ram and Grinvald, 1987; Sun and Chiu, 1999; Kukley et al., 2007). They result in buildup of axonal Ca^2+^ microdomains which are involved in triggering fast vesicular release of glutamate at synaptic-like junctions between axons and glia cells (Kukley et al., 2007; Ziskin et al., 2007). Intriguingly, peripheral axons also appear capable of releasing neurotransmitters (glutamate and acetylcholine) from their shafts at least in two experimental paradigms: (a) when nerves are dissected from an animal, placed in Ringer solution and stimulated electrically (Lissak, 1939; Vizi et al., 1983); or (b) when dissected nerves are pre-loaded with labeled neurotransmitters, (e.g., ^14^C-glutamate, tritiated choline, d-2, 3-(3)H-aspartic acid) and stimulated electrically or magnetically (Wheeler et al., 1966; DeFeudis, 1971; Weinreich and Hammerschlag, 1975; Vizi et al., 1983; Wieraszko and Ahmed, 2009). The mechanisms of neurotransmitter release from peripheral nerve axons *in situ* or *in vivo* remain largely un-investigated. But it is tempting to speculate that peripheral axons utilize similar mechanism of release as callosal and optic nerve axons, i.e., VGCCs located along axonal shafts mediate Ca^2+^ influx followed by fusion and release of neurotransmitter filled vesicles. Subsequently, released neurotransmitter may bind to its receptors on the neighboring Schwann cells. In line with this hypothesis are the recent findings in cell culture demonstrating that vesicular release of glutamate occurs along the axons of dorsal root ganglion neurons and mediates axonal-glia communication important for myelination (Wake et al., 2011, 2015). Notably, L- and N-type VGCCs expressed in peripheral nerve axons are the VGCCs subtypes which are involved in neurotransmitter release at Ribbon synapses and at conventional synapses between neurons, respectively (Catterall, 2011). Few older studies also show that axonal release in peripheral nerves resembles the axon terminal release in many respects, e.g., it depends on extracellular Ca^2+^ and is stimulated by elevated extracellular K^+^ (Dettbarn and Rosenberg, 1966; Vizi et al., 1983; Wieraszko and Ahmed, 2009). Yet, other investigators do not support these findings and suggest that the mechanism of axonal release in the peripheral nerves differs from release at synapses (Weinreich and Hammerschlag, 1975).

Finally, evidence is currently accumulating that multiple subtypes of VGCCs may contribute to injury mechanisms of central white matter axons (Fern et al., 1995; Brown et al., 2001; Tsutsui and Stys, 2013). In light of those findings it is likely that in addition to their physiological role, also VGCCs located along peripheral nerve axons may be of significance during pathological conditions, e.g., nerve injury, pain, or peripheral neuropathy.

## Author Contributions

All experiments were conducted in the laboratory of MK at the Centre for Integrative Neuroscience, University of Tübingen. MK and RB designed experiments. RB and FP performed experiments and analyzed data. MK and RB interpreted the findings, prepared the figures, and wrote the manuscript.

## Funding

This work was supported by the Deutsche Forschungsgemeinschaft (DFG) grants: KU2569/1-1 to MK, and PF574/5-1 to FP. This work was also supported by the Werner Reichardt Centre for Integrative Neuroscience (CIN) at the Eberhard Karls University of Tübingen. The CIN is an Excellence Cluster funded by the DFG within the framework of the Excellence Initiative (EXC 307).

## Conflict of Interest Statement

The authors declare that the research was conducted in the absence of any commercial or financial relationships that could be construed as a potential conflict of interest.

## Abbreviations

ACSF: artificial cerebrospinal fluid
BAPTA: 1,2-bis(o-aminophenoxy)ethane-N, N, N′, N′-tetraacetic acid
Ca^2+^: calcium
Cd^2+^: cadmium
ChAT: choline acetyltransferase
DAPI: 4′,6-diamidino-2-phenylindole
K^+^: potassium
K_d_: dissociation constant
MBP: myelin basic protein
Na^+^: sodium
NF200: neurofilament 200 kDa
OGB-1: Oregon Green BAPTA-1
PBS: phosphate-buffered saline
PFA: paraformaldehyde
RRX: rhodamine red X
TBS: tris-buffered saline
TTA-P2: 3,5-dichloro-N-[1-(2,2-dimethyl-tetrahydro-pyran-4-ylmethyl)-4-fluoro-piperidin-4-ylmethyl]-benzamide
TTX: tetrodotoxin
VGCCs: voltage-gated calcium channels.

## References

[B1] Alix J. J. Dolphin A. C. Fern R. (2008). Vesicular apparatus, including functional calcium channels, are present in developing rodent optic nerve axons and are required for normal node of Ranvier formation. J. Physiol. 586, 4069–4089. 10.1113/jphysiol.2008.155077 18599536 PMC2652192

[B2] Augustine G. J. Charlton M. P. (1986). Calcium dependence of presynaptic calcium current and post-synaptic response at the squid giant synapse. J. Physiol. 381, 619–640. 10.1113/jphysiol.1986.sp016347 2442355 PMC1182999

[B3] Bean B. P. (1985). Two kinds of calcium channels in canine atrial cells. Differences in kinetics, selectivity and pharmacology. J. Gen. Physiol. 86, 1–30. 10.1085/jgp.86.1.1 2411846 PMC2228774

[B4] Berridge M. J. Bootman M. D. Roderick H. L. (2003). Calcium signalling: dynamics, homeostasis and remodelling. Nat. Rev. Mol. Cell Biol. 4, 517–529. 10.1038/nrm1155 12838335

[B5] Borst J. G. Helmchen F. Sakmann B. (1995). Pre- and postsynaptic whole-cell recordings in the medial nucleus of the trapezoid body of the rat. J. Physiol. 489, 825–840. 10.1113/jphysiol.1995.sp021095 8788946 PMC1156851

[B6] Brown A. M. Westenbroek R. E. Catterall W. A. Ransom B. R. (2001). Axonal L-type Ca^2+^ channels and anoxic injury in rat CNS white matter. J. Neurophysiol. 85, 900–911. 11160521 10.1152/jn.2001.85.2.900

[B7] Bucher D. Goaillard J. M. (2011). Beyond faithful conduction: short-term dynamics, neuromodulation and long-term regulation of spike propagation in the axon. Prog. Neurobiol. 94, 307–346. 10.1016/j.pneurobio.2011.06.001 21708220 PMC3156869

[B8] Callewaert G. Eilers J. Konnerth A. (1996). Axonal calcium entry during fast ‘sodium’ action potentials in rat cerebellar Purkinje neurones. J. Physiol. 495, 641–647. 10.1113/jphysiol.1996.sp021622 8887772 PMC1160771

[B9] Catterall W. A. (2011). Voltage-gated calcium channels. Cold Spring Harb. Perspect. Biol. 3:a003947 10.1101/cshperspect.a003947 21746798 PMC3140680

[B10] Chan S. Y. Ochs S. Worth R. M. (1980). The requirement for calcium ions and the effect of other ions on axoplasmic transport in mammalian nerve. J. Physiol. 301, 477–504. 10.1113/jphysiol.1980.sp013219 6157806 PMC1279412

[B11] DeFeudis F. V. (1971). Effects of electrical stimulation on the efflux of L-glutamate from peripheral nerve *in vitro*. Exp. Neurol. 30, 291–296. 10.1016/s0014-4886(71)80008-0 5547254

[B12] Delvendahl I. Jablonski L. Baade C. Matveev V. Neher E. Hallermann S. (2015). Reduced endogenous Ca^2+^ buffering speeds active zone Ca^2+^ signaling. Proc. Natl. Acad. Sci. U S A 112, E3075–E3084. 10.1073/pnas.1508419112 26015575 PMC4466756

[B13] Dettbarn W. D. Rosenberg P. (1966). Effect of ions on the efflux of acetylcholine from peripheral nerve. J. Gen. Physiol. 50, 447–460. 10.1085/jgp.50.2.447 11526839 PMC2225652

[B14] Dolphin A. C. (2006). A short history of voltage-gated calcium channels. Br. J. Pharmacol. 147, S56–S62. 10.1038/sj.bjp.0706442 16402121 PMC1760727

[B15] Eberhardt M. Hoffmann T. Sauer S. K. Messlinger K. Reeh P. W. Fischer M. J. (2008). Calcitonin gene-related peptide release from intact isolated dorsal root and trigeminal ganglia. Neuropeptides 42, 311–317. 10.1016/j.npep.2008.01.002 18328558

[B16] Elliott P. Marsh S. J. Brown D. A. (1989). Inhibition of Ca-spikes in rat preganglionic cervical sympathetic nerves by sympathomimetic amines. Br. J. Pharmacol. 96, 65–76. 10.1111/j.1476-5381.1989.tb11785.x 2538183 PMC1854318

[B17] Ermolyuk Y. S. Alder F. G. Surges R. Pavlov I. Y. Timofeeva Y. Kullmann D. M.. (2013). Differential triggering of spontaneous glutamate release by P/Q-, N- and R-type Ca^2+^ channels. Nat. Neurosci. 16, 1754–1763. 10.1038/nn.3563 24185424 PMC4176737

[B18] Fern R. Ransom B. R. Waxman S. G. (1995). Voltage-gated calcium channels in CNS white matter: role in anoxic injury. J. Neurophysiol. 74, 369–377. 7472338 10.1152/jn.1995.74.1.369

[B19] Forti L. Pouzat C. Llano I. (2000). Action potential-evoked Ca^2+^ signals and calcium channels in axons of developing rat cerebellar interneurones. J. Physiol. 527, 33–48. 10.1111/j.1469-7793.2000.00033.x 10944168 PMC2270052

[B20] François A. Schüetter N. Laffray S. Sanguesa J. Pizzoccaro A. Dubel S.. (2015). The low-threshold calcium channel Cav3.2 determines low-threshold mechanoreceptor function. Cell Rep. [Epub ahead of print]. 10.1016/j.celrep.2014.12.042 25600872

[B21] Huguenard J. R. (1996). Low-threshold calcium currents in central nervous system neurons. Annu. Rev. Physiol. 58, 329–348. 10.1146/annurev.ph.58.030196.001553 8815798

[B22] Jackson V. M. Trout S. J. Brain K. L. Cunnane T. C. (2001). Characterization of action potential-evoked calcium transients in mouse postganglionic sympathetic axon bundles. J. Physiol. 537, 3–16. 10.1111/j.1469-7793.2001.0003k.x 11711556 PMC2278936

[B23] Jirounek P. Chardonnens E. Brunet P. C. (1991). After potentials in nonmyelinated nerve fibers. J. Neurophysiol. 65, 860–873. 2051207 10.1152/jn.1991.65.4.860

[B24] Kandel E. R. Schwartz J. H. Jessell T. M. (2000). Principles of Neural Science. New York: McGraw-Hill Companies, Inc.

[B25] Katz E. Ferro P. A. Weisz G. Uchitel O. D. (1996). Calcium channels involved in synaptic transmission at the mature and regenerating mouse neuromuscular junction. J. Physiol. 497, 687–697. 10.1113/jphysiol.1996.sp021800 9003554 PMC1160965

[B26] Kinkelin I. Brocker E. B. Koltzenburg M. Carlton S. M. (2000). Localization of ionotropic glutamate receptors in peripheral axons of human skin. Neurosci. Lett. 283, 149–152. 10.1016/s0304-3940(00)00944-7 10739897

[B27] Koester H. J. Sakmann B. (2000). Calcium dynamics associated with action potentials in single nerve terminals of pyramidal cells in layer 2/3 of the young rat neocortex. J. Physiol. 529, 625–646. 10.1111/j.1469-7793.2000.00625.x 11118494 PMC2270226

[B28] Kostyuk P. G. Veselovsky N. S. Tsyndrenko A. Y. (1981). Ionic currents in the somatic membrane of rat dorsal root ganglion neurons-I. Sodium currents. Neuroscience 6, 2423–2430. 10.1016/0306-4522(81)90088-9 6275294

[B29] Kukley M. Capetillo-Zarate E. Dietrich D. (2007). Vesicular glutamate release from axons in white matter. Nat. Neurosci. 10, 311–320. 10.1038/nn1850 17293860

[B30] Lehning E. J. Doshi R. Isaksson N. Stys P. K. LoPachin R. M. Jr. (1996). Mechanisms of injury-induced calcium entry into peripheral nerve myelinated axons: role of reverse sodium-calcium exchange. J. Neurochem. 66, 493–500. 10.1046/j.1471-4159.1996.66020493.x 8592118

[B31] Lev-Ram V. Grinvald A. (1987). Activity-dependent calcium transients in central nervous system myelinated axons revealed by the calcium indicator Fura-2. Biophys. J. 52, 571–576. 10.1016/s0006-3495(87)83246-0 3676438 PMC1330047

[B32] Lissak K. (1939). Liberation of acetylcholine and adrenaline by stimulating isolated nerves. Am. J. Physiol. 127, 263–271.

[B33] Lüscher C. Lipp P. Lüscher H. R. Niggli E. (1996). Control of action potential propagation by intracellular Ca^2+^ in cultured rat dorsal root ganglion cells. J. Physiol. 490, 319–324. 10.1113/jphysiol.1996.sp021146 8821131 PMC1158671

[B34] Matute C. (2010). Calcium dyshomeostasis in white matter pathology. Cell Calcium 47, 150–157. 10.1016/j.ceca.2009.12.004 20036774

[B35] Mayer C. Quasthoff S. Grafe P. (1999). Confocal imaging reveals activity-dependent intracellular Ca^2+^ transients in nociceptive human C fibres. Pain 81, 317–322. 10.1016/s0304-3959(99)00015-9 10431719

[B36] Mintz I. M. Sabatini B. L. Regehr W. G. (1995). Calcium control of transmitter release at a cerebellar synapse. Neuron 15, 675–688. 10.1016/0896-6273(95)90155-8 7546746

[B37] Nudler S. Piriz J. Urbano F. J. Rosato-Siri M. D. Renteria E. S. Uchitel O. D. (2003). Ca^2+^ channels and synaptic transmission at the adult, neonatal and P/Q-type deficient neuromuscular junction. Ann. N Y Acad. Sci. 998, 11–17. 10.1196/annals.1254.003 14592858

[B38] Ouardouz M. Nikolaeva M. A. Coderre E. Zamponi G. W. McRory J. E. Trapp B. D.. (2003). Depolarization-induced Ca^2+^ release in ischemic spinal cord white matter involves L-type Ca^2+^ channel activation of ryanodine receptors. Neuron 40, 53–63. 10.1016/j.neuron.2003.08.016 14527433

[B39] Quasthoff S. Adelsberger H. Grosskreutz J. Arzberger T. Schroder J. M. (1996). Immunohistochemical and electrophysiological evidence for omega-conotoxin-sensitive calcium channels in unmyelinated C-fibres of biopsied human sural nerve. Brain Res. 723, 29–36. 10.1016/0006-8993(96)00186-2 8813379

[B40] Quasthoff S. Grosskreutz J. Schröder J. M. Schneider U. Grafe P. (1995). Calcium potentials and tetrodotoxin-resistant sodium potentials in unmyelinated C fibres of biopsied human sural nerve. Neuroscience 69, 955–965. 10.1016/0306-4522(95)00307-5 8596662

[B41] Regehr W. G. (2000). “Monitoring presynaptic calcium dynamics with membrane-permeant indicators,” in Imaging Neurons. A Laboratory Manual, eds Yuste R. Lanni F. Konnerth A. (New York, NY: Cold Spring Harbor Laboratory Press), 37.1–37.11.

[B42] Regehr W. G. Atluri P. P. (1995). Calcium transients in cerebellar granule cell presynaptic terminals. Biophys. J. 68, 2156–2170. 10.1016/s0006-3495(95)80398-x 7612860 PMC1282121

[B43] Sargoy A. Sun X. Barnes S. Brecha N. C. (2014). Differential calcium signaling mediated by voltage-gated calcium channels in rat retinal ganglion cells and their unmyelinated axons. PLoS One 9:e84507. 10.1371/journal.pone.0084507 24416240 PMC3885580

[B44] Spitzer M. J. Reeh P. W. Sauer S. K. (2008). Mechanisms of potassium- and capsaicin-induced axonal calcitonin gene-related peptide release: involvement of L- and T-type calcium channels and TRPV1 but not sodium channels. Neuroscience 151, 836–842. 10.1016/j.neuroscience.2007.10.030 18178321

[B45] Sun B. B. Chiu S. Y. (1999). N-type calcium channels and their regulation by GABAB receptors in axons of neonatal rat optic nerve. J. Neurosci. 19, 5185–5194. 10377330 10.1523/JNEUROSCI.19-13-05185.1999 PMC6782304

[B46] Swandulla D. Hans M. Zipser K. Augustine G. J. (1991). Role of residual calcium in synaptic depression and posttetanic potentiation: fast and slow calcium signaling in nerve terminals. Neuron 7, 915–926. 10.1016/0896-6273(91)90337-y 1662519

[B47] Thaxton C. Bott M. Walker B. Sparrow N. A. Lambert S. Fernandez-Valle C. (2011). Schwannomin/merlin promotes Schwann cell elongation and influences myelin segment length. Mol. Cell. Neurosci. 47, 1–9. 10.1016/j.mcn.2010.12.006 21182951 PMC3129596

[B48] Tsutsui S. Stys P. K. (2013). Metabolic injury to axons and myelin. Exp. Neurol. 246, 26–34. 10.1016/j.expneurol.2012.04.016 22569104

[B49] Villegas R. Martinez N. W. Lillo J. Pihan P. Hernandez D. Twiss J. L.. (2014). Calcium release from intra-axonal endoplasmic reticulum leads to axon degeneration through mitochondrial dysfunction. J. Neurosci. 34, 7179–7189. 10.1523/JNEUROSCI.4784-13.2014 24849352 PMC4028495

[B50] Vizi E. S. Gyires K. Somogyi G. T. Ungváry G. (1983). Evidence that transmitter can be released from regions of the nerve cell other than presynaptic axon terminal: axonal release of acetylcholine without modulation. Neuroscience 10, 967–972. 10.1016/0306-4522(83)90234-8 6646439

[B51] Wächtler J. Mayer C. Grafe P. (1998). Activity-dependent intracellular Ca^2+^ transients in unmyelinated nerve fibres of the isolated adult rat vagus nerve. Pflugers Arch. 435, 678–686. 10.1007/s004240050569 9479021

[B52] Wake H. Lee P. R. Fields R. D. (2011). Control of local protein synthesis and initial events in myelination by action potentials. Science 333, 1647–1651. 10.1126/science.1206998 21817014 PMC3482340

[B53] Wake H. Ortiz F. C. Woo D. H. Lee P. R. Angulo M. C. Fields R. D. (2015). Nonsynaptic junctions on myelinating glia promote preferential myelination of electrically active axons. Nat. Commun. 6:7844. 10.1038/ncomms8844 26238238 PMC4532789

[B54] Wang L. Y. Augustine G. J. (2015). Presynaptic nanodomains: a tale of two synapses. Front. Cell. Neurosci. 8:455. 10.3389/fncel.2014.00455 25674049 PMC4306312

[B55] Weinreich D. Hammerschlag R. (1975). Nerve impulse-enhanced release of amino acids from non-synaptic regions of peripheral and central nerve trunks of bullfrog. Brain Res. 84, 137–142. 10.1016/0006-8993(75)90807-0 234273

[B56] Wheeler D. D. Boyarsky L. L. Brooks W. H. (1966). The release of amino acids from nerve during stimulation. J. Cell. Physiol. 67, 141–147. 10.1002/jcp.1040670116 5940405

[B57] Wieraszko A. Ahmed Z. (2009). Axonal release of glutamate analog, d-2,3–3H-Aspartic acid and l-14C-proline from segments of sciatic nerve following electrical and magnetic stimulation. Neurosci. Lett. 458, 19–22. 10.1016/j.neulet.2009.04.024 19442870

[B58] Yuan Y. Jiang C. Y. Xu H. Sun Y. Hu F. F. Bian J. C.. (2013). Cadmium-induced apoptosis in primary rat cerebral cortical neurons culture is mediated by a calcium signaling pathway. PLoS One 8:e64330. 10.1371/journal.pone.0064330 23741317 PMC3669330

[B59] Zhang C. L. Wilson J. A. Williams J. Chiu S. Y. (2006). Action potentials induce uniform calcium influx in mammalian myelinated optic nerves. J. Neurophysiol. 96, 695–709. 10.1152/jn.00083.2006 16835363

[B60] Ziskin J. L. Nishiyama A. Rubio M. Fukaya M. Bergles D. E. (2007). Vesicular release of glutamate from unmyelinated axons in white matter. Nat. Neurosci. 10, 321–330. 10.1038/nn1854 17293857 PMC2140234

